# Fibromyalgia: Chronic Pain Due to a Blood Dysfunction?

**DOI:** 10.3390/ijms26094153

**Published:** 2025-04-27

**Authors:** Anna Maria Aloisi, Ilenia Casini

**Affiliations:** Stress and Pain Neurophysiology Laboratory, Department of Medicine, Surgery and Neuroscience University of Siena, 53100 Siena, Italy; ilenia.casini2@unisi.it

**Keywords:** chronic pain, blood, vessels, ANS, 5-HT, hypoxia, gonadal hormones

## Abstract

Fibromyalgia (FM) is a common chronic disorder with chronic pain. FM generally affects all ages and occurs more commonly in women. The cause of FM remains undefined, but a number of factors suggest the cardiovascular system and the blood in particular as contributors to its occurrence and maintenance. Hemograms and other blood indexes often show high percentages of values at the ‘normal’, low, or high limits and several values outside of the ‘normal’ ranges. On the other hand, vessels regulate blood arrival to tissues depending on many internal and external factors. Both aspects can interfere with tissue oxygenation and then with the numerous consequences induced by hypoxia. In this narrative review, efforts were made to highlight factors that are potentially able to affect oxygen arrival in cells, as well as other factors related to blood elements that can play a role in the chronic pain experienced by FM patients. Data strongly indicate that most of the symptoms commonly present in FM patients can find their physio-pathological basis in the blood, suggesting blood-related interventions in these patients.

## 1. Introduction

Body functions need energy, i.e., ATP. To allow the production of ATP, cells need nutrients and oxygen (O_2_). As for nutrients, although cells are able to transform all food elements into glucose, O_2_ has to arrive through the circulation. Its use can be delayed but not avoided. Respiration consists of the ability to bring O_2_ to ALL cells in the body. O_2_ availability needs to follow tissue requirements. If the supply is not sufficient (hypoxia), localized or generalized consequences can occur, such as inflammation and the production of ROS and cytokines. All these conditions are known to be directly and/or indirectly related to pain occurrence and chronicization.

Fibromyalgia (FM) is a chronic painful condition affecting 2–3% of the population, mostly women [1]. The painful condition is characterized by diffuse musculoskeletal pain. Its origin is still not clearly defined, and drugs are not useful. FM includes not only pain but many other symptoms/signs, such as fatigue, depression, muscle pain, and gut disorders, all possibly related to blood features. The high sexual ‘preference’ towards females has something to do with the female sex, and the cardiovascular system shows important sex differences. Many cardiovascular features are related to gonadal hormones, particularly estrogens. In women, the concentration of estrogens changes daily and drastically decreases with menopause.

Looking at fibromyalgia (FM) patients [2], it was clear that although most had blood parameters within the ‘normal’ range, several values were very close to the higher or lower limits. For instance, about 50% of red blood cell (RBC) values were ‘normally’ high, close to or higher than 5 million/mm^3^, with the hematocrit (HT) close to or higher than 44%. Thus, many studies carried out to evaluate different aspects of the vascular system, from vessel function to the number of blood cells, reported both functional and morphological abnormalities in FM patients. For instance, fewer capillaries in the nail fold and significantly more capillary dilations were found in FM patients than in controls, together with lower peripheral blood flow [3,4,5]. In another study, the density of capillaries per mm in FM patients was lower than in controls but still in the normal range [6]. Regarding RBCs, the surface area/volume ratio, which is essential for maintaining the cells’ oxygen-carrying capacity, was found to be higher in FM patients than in controls [7]. Al-Allaf et al. [8] found higher plasma viscosity values in FM patients than in controls, while Arihan et al. [9] found no differences in the same parameters. Moreover, hemoglobin (Hb) values in FM patients were lower than in controls [9], but in another study [10] Hb values in the FM group were found to be significantly higher when compared to controls, while Mader et al. [11] reported no differences. Rus et al. [12] reported significantly higher RBC and HT values in FM women than in controls, and lower mean corpuscular Hb concentration (MCHC) values than in healthy volunteers, suggesting that the RBCs of FM patients may contain lower concentrations of Hb than those of the healthy subjects. Low MCHC values in FM patients may lead to impaired tissue oxygen delivery and ultimately to fatigue, one of the most characteristic symptoms for patients diagnosed with FM. In the same study [12], women with FM had significantly higher platelet counts than healthy women. Alves et al. [13] observed a decrease in Hb, HT, and testosterone in the serum of FM patients compared to the control group. Telli and Ozdemir [14] found a significant correlation between a reduction in Hb and ferritin and an increase in pain intensity (VAS) in patients with chronic pain. Molina et al. [15] observed an increase in platelets and RBCs in FM subjects. In a recent review [1], many other blood-related differences between FM subjects and controls are reported regarding nutrients and functional elements.

The reported evidence suggests that, in FM patients, something does not allow the regular arrival of oxygen to the cells and/or the regular production of energy. Some possible aspects are discussed in the present narrative review.

## 2. Hypoxia

A possible involvement of hypoxia in FM pain was suggested by Fassbender [16], who identified muscle alterations due to local hypoxia. This hypothesis was further tested [5,17,18], and the evidence of a pathological distribution of muscle surface oxygenation was confirmed. A correlation was found between pain and reduced blood flow [19]. In this case, local muscle pain was related to local temporary hypoxia. Later, in FM patients, an increased concentration of RBCs, decreased RBC velocity, and a consequent decrease in the flux of RBCs were found in the skin above the tender points. Indeed, a lower temperature was recorded above the tender points. All the data suggest the presence of lower blood flow due to vessel vasoconstriction (Table 1).

Vasoconstriction is a common physiological condition and can be induced by different substances able to induce smooth muscle contraction, together with a fall in O_2_ tissue pressure, in the affected district [20]. In all cases, there is a decrease in blood flow and a consequent imbalance between oxygen supply and demand; indeed, in FM, a decrease in ATP and phosphocreatine in the tender muscle was described [21].

Local hypoxia, if long-lasting, will induce not only a decrease in the concentration of high-energy phosphate but also morphological changes and pain [5]. Importantly, to be considered in FM, there is the possibility that long-lasting hypoxia can induce structural changes not only in muscle cells but also in nerves, leading to neuroimpairment, i.e., damage to the small fibers [22]. Indeed, the presence of small fiber alterations was shown in FM patients [23,24]. As for the cause of this condition, only slight vessel morphological abnormalities were described [3], suggesting the abnormal regulation of capillary blood flow rather than morphological changes in the capillaries.

Hypoxia can be accompanied by a higher RBC count and a high hematocrit (HT); these conditions, apparently positive, allow cell adherence to the vascular bed, slowing the passage of blood. This event promotes hypoxia and the consequent jamming of the post-capillary venules.

RBCs, platelets, and neutrophils also mediate intercellular interactions among themselves to form aggregates in the flow [25]. Such interactions are mediated primarily by P-selectin, which is expressed on endothelial cells and platelets in response to inflammation. Recently, it was shown that neutrophil–platelet aggregation can be mediated by platelet-derived exosomes carrying IL-1B and caspase-1 [26]. Exosomes are extracellular microvesicles released by numerous cell types into biological fluids and are known to be involved in regulatory, physiological, and pathophysiological processes, modifying functional processes and the functional phenotype of target cells [27]. Mast cell-derived exosomes have been shown to activate endothelial cells to secrete plasminogen activation inhibitor-1 (PAI-1), which is able to delay aggregate resolution [28].

On the whole, all these factors can contribute to hypoxia, a condition that can be present notwithstanding the high number of circulating RBCs.

## 3. Blood Volume

In humans, blood represents 7% of the body weight, i.e., about 5 liters. This volume is not sufficient to completely fill all vessels in the body; blood needs to be re-distributed moment-by-moment in the different districts to allow O_2_ and nutrients to reach the gut or muscles, the skin or gut, etc. For instance, the difficulty of practicing physical activities during digestion is well-known. Water represents more than 90% of the blood’s volume and is continuously taken/given to interstitial spaces. The volume of water present in the blood can decrease significantly if needed in physiological processes such as digestion; depending on food osmolarity, liters of water can be transferred to the gut to help digestive activity. In addition, water is continuously lost also due to kidney activity (from 400 to 1500 mL per day), sweating, and respiration. All these physiological events can lead to low water levels in the blood, with increases in osmolality and viscosity if not adequately consumed during the day. In many subjects, including a high number of FM patients, diarrhea plays an important role [2], since it leads to high volumes of water loss with the feces. Diarrhea can also be induced by food intolerances and/or drug consumption able to alter gut functions. FM patients were often found to be hyperglycemic [12], a condition that could cause significant polyuria.

As for water consumption, it is generally known that it is mandatory to drink 1.5 L a day. However, the determination of water consumption carried out in our experiments [2,29] confirms the scarcity of drinking in the majority of pain patients. Moreover, many aged subjects report a lack of thirst.

On the whole, this rarely considered parameter, needs to be included as a possible important factor in chronic pain occurrence.

## 4. Blood Vessels

Blood reaches tissues and cells through vessels ranging from large to very small in caliber. Blood vessels’ smooth muscle walls respond to many influences that, depending on their caliber, regulate and distribute peripheral perfusion. O_2_ delivery to body areas can be strongly affected by a number of substances released/produced under different conditions able to affect smooth muscle contraction, causing vasorelaxation or vasoconstriction [30].

Vessels can be relaxed by factors such as nitric oxide (NO), prostacyclin-2 (PGI2), and endothelium-derived hyperpolarizing factor (EDHF). In particular, NO is produced in the endothelium by eNOS synthase; NO has a very short half-life and acts on smooth muscle cells, causing Ca++ sequestration and then smooth muscle relaxation [31]. NO production is highly sensitive to several factors, including gonadal hormones. Indeed, both estradiol and testosterone increase eNOS activity and NO bioavailability [32]. On the other hand, several substances can induce vasoconstriction. Firstly, neuronal and humoral noradrenaline (NA) and adrenaline (A) [33] are major vasoconstrictors (but also vasorelaxants) in the entire vascular tree through their receptors (α and β) located in muscles and endothelial cells. In particular, α2 receptors are predominantly in control of sympathetic vasoconstriction [34]. Stress is the main factor that activates their massive release. During a stress response with high secretion of noradrenaline, the blood flow through the muscle can decrease to about 25% of the normal rate, inducing certain levels of hypoxia [35]. Vasospastic symptoms occur in about 30% of patients with primary FM [36]. Bennet et al. [37] found an increased density of adrenergic receptors in FM patients, which predisposes them to cold- and emotion-induced vasospasms. It is well-known that most women start to suffer chronic pain after a period of great stress and/or an infection [1].

Interestingly, sex differences have been shown in the vasodilatory (β-receptor-mediated) responses of male and female blood vessels towards adrenergic stimuli. Indeed, β-adrenergic stimulation increases forearm blood flow more profoundly in women compared to men. Sex differences were found also in endothelial β-adrenoceptors since the aorta expresses more endothelial β1 and β3 adrenoceptors in female rats than in male rats [38]. These receptors are estrogen-dependent; thus, their presence/activity will follow estrogen fluctuations and/or drastic decreases after menopause.

Angiotensin II and vasopressin are both strong vasoconstrictors that decrease blood flow during low water availability in the blood. Angiotensin II acts on AT1 receptors, and its main functions are to induce aldosterone production by the adrenals (to increase Na+ reabsorption), stimulate thirst via the hypothalamus, and cause general vasoconstriction. Indeed, it is a strong vasoconstrictor, acting especially on vessels with little resistance. Angiotensin II is produced in the lung parenchyma and in the endothelium in general from angiotensin I due to the presence of angiotensin-converting enzyme (ACE) in these cells. Several studies reported a detrimental role of angiotensin II towards endothelial functions, particularly by activating endothelial mechanisms suppressing NO production and bioavailability. Angiotensin II was found to increase basal ROS production and aggravate oxidative stress [39].

Vasopressin, the antidiuretic hormone, acts on V1 and V2 receptors that, in addition to allowing water reabsorption in the kidney, induce strong general vasoconstriction activity.

Another important factor that is able to significantly reduce blood flow is endothelin-1 (ET-1). It is a potent vasoconstrictor mediating its effect via ETα and ETβ receptors, which are both expressed on vascular smooth muscles. Under baseline physiological conditions, ET-1 formation is low. In endothelial dysfunction, ET-1 production is enhanced while NO availability is reduced, thus reducing blood flow. In addition, ET-1 may also promote vascular smooth muscle cell hypertrophy and proliferation and exert pro-inflammatory actions in vessels.

All these activities are regulated by reflexes and are not acknowledged by the patients. A blood perfusion decrease can be considered an important aspect able to induce hypoxia and diffuse pain.

## 5. Serotonin

**Figure 1:**
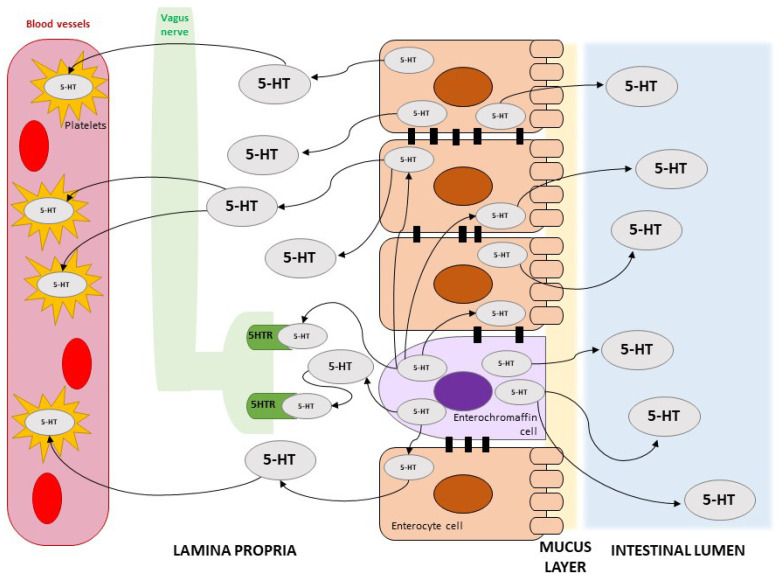
Schematic representation of the presence of serotonin (5-HT) in the gut and blood, as well as possible interactions.

Serotonin (5-HT) acts to modulate different aspects of body functions and is an important factor able to affect blood flow in many compartments (Figure 1). 5-HT is produced mainly by the enterochromaffin cells of the gut (95%), where it has functions in microbiota health, gut motility, and vagus nerve modulation, among others. 5-HT antagonists and/or reuptake blockers are commonly used to treat gut and nervous system disorders [40,41,42,43]. Platelets circulating in the gut vessels are able to take up 5-HT [44,45]. Thus, platelets serve as a systemic reservoir of 5-HT and are able to deliver it to remote peripheral tissues [46]. Platelets can release 5-HT once in contact with altered vessels (i.e., during inflammatory states). Indeed, inflammation can change their features, increasing wall adhesion towards blood cells, with a consequent risk of thrombosis [47]. Moreover, platelets express 5-HT receptors that, when stimulated, promote platelet aggregation [48].

A low-grade inflammatory state is a common condition mainly caused by gut disorders. Inflammation is accompanied by the release of inflammatory substances (i.e., cytokines) that can affect vessels and the interaction between platelets and vessels. On the other hand, once the gut is inflamed, it can increase circulating 5-HT [1], which acts on platelets and induces hypercoagulability, rendering these subjects susceptible to thrombotic events and hypoxia. This is in agreement with clinical data suggesting that several categories of GI disorders are associated with an increased risk of ischemic stroke [49].

Another hypothesis related to 5-HT includes the fact that 5-HT directly induces vasoconstriction in large arteries and veins and enhances the contractile effect of other vasoconstrictors, such as angiotensin II and histamine [50,51]. In contrast, in arterioles, 5-HT exerts a vasodilatory effect via the 5-HT receptor, NO release, and vascular smooth muscle relaxation [52]. Thus, since several investigators have reported low serum 5-HT levels in FM patients as compared to the general population [1], these low 5-HT levels could be the reason for the lack of vasodilatory effect at the arteriole level with decreases in local tissue flow.

## 6. Steroid Hormones

Gonadal hormones are hormones secreted by the gonads, adrenals, and all tissues in which enzymes are present that are able to synthesize them, including the CNS (neurosteroids) and the fat, where aromatase transforms testosterone into estradiol [53]. Estrogen shows higher levels in women, although its presence is also significant in men. Estrogen’s protective effect on the cardiovascular system of women is well-known, particularly due to the clear clinical evidence of lower cardiovascular accidents in women than in men until menopause. After menopause, the ovaries produce less estrogen and cardiovascular accidents become the most common cause of death in women [54].

Estrogens are involved in many aspects of cardiovascular regulation, such as blood lipid regulation, vasodilation, and antioxidant and anti-inflammatory effects [55]. It was reported that younger women of reproductive age have lower amounts of fibrinogen binding to platelets than menopausal women [56]; moreover, estrogen has been shown to have an anti-atherosclerotic effect by modulating platelet activity through the release of vasodilator substances such as NO [57]. A similar effect is obtained through estrogen inhibiting Ca++ entry from the extracellular space in smooth muscles, since Ca++ is fundamental for muscle contraction [58].

Synthesis of NO can be altered after estrogen decrease due to physiological changes (menopause), surgery (ovary exportation), stress events able to decrease HPG activity, and drug-induced endocrinopathies [59]. The decrease in NO production/bioavailability and the consequent overall loss of endothelial protective effects, as well as the maintenance of vascular homeostasis, are considered prime hallmarks of endothelial dysfunction. An increase in vessel tone is an almost instantaneous result of decreased endothelial NO production. Indeed, since NO has an important autocrine inhibitory effect on ET-1 synthesis, whereas ET-1, in turn, inhibits eNOS activity, less NO production may increase the release of ET-1 from endothelial cells, further increasing vascular tone.

Platelet activity seems to be sensitive to hormonal changes, which may in part be explained by the presence of estrogen receptors on platelet membranes [60,61]. Estrogens were found to inhibit platelet aggregation at high levels [62], although platelet adhesion to endothelial cells was increased after estrogen treatment [63]. Interestingly, in subjects with low levels of estrogens, higher levels of circulating microparticles from platelets, granulocytes, monocytes, and endothelial cells were found [64]. These platelet-derived microparticles, which are released from activated platelets, have been described to promote inflammation and thrombosis by inducing the release of inflammatory mediators, such as TNFα and IL1β, from monocytes and by expressing tissue factors, which activate factor VII and, subsequently, the extrinsic coagulation pathway [65,66].

Another interesting hypothesis involving NO is related to its high levels in the circulating blood and cerebrospinal fluid of FM patients [1]. Prolonged release of high levels of NO in people with FM may induce accelerated apoptosis and abnormal cell death in muscle tissue, which might contribute to the structural and metabolic defects identified in the muscles of patients with FM [67].

## 7. Inflammation

Adhesion of RBCs to the vascular endothelium is an essential precursor facilitating occlusion in vivo. Such interactions are not only mediated by the expression of adhesion proteins on activated RBCs and endothelial cells but also through complex cell–cell interactions among RBC, leukocytes, platelets, and endothelin [68]. The presence of an inflammatory condition in FM is supported by a higher erythrocyte sedimentation rate (ESR) than in controls [69,70]. ESR is a non-specific marker whose elevation may involve inflammatory processes.

The rate of blood flow decreased when leukocytes were primed with TNFα, indicating the role of inflammation in vascular stasis. Neutrophils secrete cytokines, chemokines, and enzymes, including neutrophil elastase, matrix metalloprotein 9 (MMP9), and vascular endothelial growth factor (VEGF) [71]. Lymphocytes, in turn, constitute an important component of the host’s immune system [72]. The neutrophil–lymphocyte ratio (NLR) is a useful marker in the assessment of inflammatory responses [73]. We recently observed [29] that, although most of the women were within the normal range, several subjects (12%, *n* = 14) had NLR values lower (<1) or higher (>2, 6%, *n* = 8) than normal, suggesting conditions deserving attention. In the same population, the platelet-to-lymphocyte ratio (PLR) was found to be moderately high/high in more than 50% of FM patients. SIRI, the systemic inflammatory index investigated in rheumatoid arthritis [74], was found to be higher in more than 30% of the subjects, suggesting the presence of a significant inflammatory state in these patients. The NLR and PLR indexes are recognized as accessible and affordable markers due to the hemogram tests routinely conducted in patients [75,76]. No studies have investigated the role of SIRI in FM patients. SIRI is a new and more comprehensive marker based on the composition ratio of peripheral blood neutrophil, monocyte, and lymphocyte counts [77,78]. Subjects with a high SIRI level had higher levels of neutrophils and monocytes and lower levels of lymphocytes. Thus, SIRI can be considered a more comprehensive inflammatory indicator based on the composition ratio of subgroups of blood cells.

## 8. Conclusions

FM is too often considered an untreatable condition with bad psychological outcomes in patients, mostly women. Focusing attention on the blood can help these subjects. Blood is a dynamic tissue that is continuously adapting to the body’s requirements. Its temporary deficiency can induce terrible pain even in healthy subjects. FM patients can suffer from this condition in a chronic form, with subtle and diffuse variations in tissue perfusion. The use of analgesics will only increase these conditions through chronic inflammatory states induced in the gut.

The parameters discussed in the present review highlight several factors able to influence the arrival of blood to tissues in good amounts to avoid hypoxia. Sufficient water consumption, as well as attention to chronic subclinical inflammation (i.e., from the gut), can help to decrease and cure these conditions.

## Figures and Tables

**Table 1:** Summary of the main causes able to induce hypoxia.

Tissue	Altered Function	Cause/Substances
Blood	➢ Decrease in oxygen arrival to the tissue	➢ Anemia ➢ Polycythemia
Blood Volume	➢ Decrease in the water content of the blood	➢ Low water intake ➢ Increase in water lost: - Diarrhea - Hyperglycemia
Blood Vessels	➢ Vasoconstriction	➢ Autonomic nervous system: - Adrenaline - Noradrenaline ➢ Angiotensin II ➢ Vasopressin ➢ Serotonin

## Abbreviations

The following abbreviations are used in this manuscript:

5-HT	serotonin
ANS	autonomic nervous system
ATP	adenosine triphosphate
CNS	central nervous system
ESR	erythrocyte sedimentation rate
FM	Fibromyalgia
Hb	Hemoglobin
HT	hematocrit
IL	Interleukin
MCHC	mean corpuscular hemoglobin concentration
NLR	neutrophil leukocyte ratio
NO	nitric oxide
O_2_	oxygen
PLR	platelet leukocyte ratio
RBC	red blood cell
SIRI	systemic inflammatory index
TNF	tumor necrosis factor
